# Human *levator veli palatini* muscle: a novel source of mesenchymal stromal cells for use in the rehabilitation of patients with congenital craniofacial malformations

**DOI:** 10.1186/s13287-020-02017-7

**Published:** 2020-11-25

**Authors:** Daniela Franco Bueno, Gerson Shigueru Kabayashi, Carla Cristina Gomes Pinheiro, Daniela Y. S. Tanikawa, Cassio Eduardo Raposo-Amaral, Diogenes Laercio Rocha, José Ricardo Muniz Ferreira, Yoichiro Shibuya, Akishige Hokugo, Reza Jarrahy, Patricia A. ZuK, Maria Rita Passos-Bueno

**Affiliations:** 1 Hospital Sírio-Libanês, Instituto de Ensino e Pesquisa, São Paulo, SP Brazil; 2 Hospital Municipal Infantil Menino Jesus, São Paulo, SP Brazil; 3 Universidade de São Paulo – USP, Instituto de Biociências, Centro de Pesquisa sobre o Genoma Humano e Células-Tronco, São Paulo, SP Brazil; 4 Hospital SOBRAPAR, Campinas, SP Brazil; 5 Instituto Militar de Engenharia (IME), Departamento de Ciências de Materiais, Programa de Pós-graduação em Ciências de Materiais, Rio de Janeiro, RJ Brazil; 6 Division of Plastic and Reconstructive Surgery, David Geffen School of Medicine, University of California Los Angeles (UCLA), Los Angeles, CA USA

**Keywords:** Bone reconstruction, Mesenchymal stromal cells, *Levator veli palatini* muscle, Osteogenic differentiation, Scaffold, Craniofacial malformations

## Abstract

**Background:**

Bone reconstruction in congenital craniofacial differences, which affect about 2–3% of newborns, has long been the focus of intensive research in the field of bone tissue engineering. The possibility of using mesenchymal stromal cells in regenerative medicine protocols has opened a new field of investigation aimed at finding optimal sources of multipotent cells that can be isolated via non-invasive procedures. In this study, we analyzed whether *levator veli palatini* muscle fragments, which can be readily obtained in non-invasive manner during palatoplasty in cleft palate patients, represent a novel source of MSCs with osteogenic potential.

**Methods:**

We obtained *levator veli palatini* muscle fragments (3–5 mm^3^), during surgical repair of cleft palate in 5 unrelated patients. Mesenchymal stromal cells were isolated from the muscle using a pre-plating technique and other standard practices. The multipotent nature of the isolated stromal cells was demonstrated via flow cytometry analysis and by induction along osteogenic, adipogenic, and chondrogenic differentiation pathways. To demonstrate the osteogenic potential of these cells in vivo, they were used to reconstruct a critical-sized full-thickness calvarial defect model in immunocompetent rats.

**Results:**

Flow cytometry analysis showed that the isolated stromal cells were positive for mesenchymal stem cell antigens (CD29, CD44, CD73, CD90, and CD105) and negative for hematopoietic (CD34 and CD45) or endothelial cell markers (CD31). The cells successfully underwent osteogenic, chondrogenic, and adipogenic cell differentiation under appropriate cell culture conditions. Calvarial defects treated with CellCeram™ scaffolds seeded with the isolated *levator veli palatini* muscle cells showed greater bone healing compared to defects treated with acellular scaffolds.

**Conclusion:**

Cells derived from *levator veli palatini* muscle have phenotypic characteristics similar to other mesenchymal stromal cells, both in vitro and in vivo. Our findings suggest that these cells may have clinical relevance in the surgical rehabilitation of patients with cleft palate and other craniofacial anomalies characterized by significant bone deficit.

**Supplementary information:**

The online version contains supplementary material available at 10.1186/s13287-020-02017-7.

## Introduction

The therapeutic potential of mesenchymal stromal cells in bone tissue engineering is promising, as their use may allow for the reconstruction of complex bone defects without the need for associated donor site morbidity, which is a distinct limitation when autologous bone grafts are used. It is expected that, once effective and uniform protocols are adapted clinically, bone tissue engineering will be used to treat a wide variety of conditions that present with bone deficit as a primary condition, including congenital malformations, or in the management of patients with secondary bone loss, as in the setting of trauma, oncologic resection, or osteoporosis.

Bone reconstruction in craniofacial diseases, which affect about 2–3% of newborns, has historically been the focus of intensive research [1]. Due to its high incidence rate, estimated to occur in approximately 1:2500 live births [2, 3], cleft palate (CP) stands out as one of the most intensively researched malformations.

In approximately 50% of cases, CP occurs as an isolated entity, while the remainder of cases are associated with various syndromes in which other structures are affected [4]. In these syndromic cases, patients may exhibit other facial bone malformations that require surgical correction, as in the case of Treacher-Collins syndrome [5] and Goldenhar syndrome [6]. The current “gold standard” approaches to facial skeletal reconstruction in these patient populations include the use of autogenous bone grafts and distraction osteogenesis [5, 6]. However, the benefits of these surgical procedures may be offset by complications such as donor site morbidity, post-surgical reabsorption, and infection [7, 8]. To circumvent these problems, researchers have focused on the development of bone tissue engineering strategies using various combinations of osteogenic materials, growth factors, and stem cells that may offer alternative methods with comparatively minimal or no donor site morbidity and lower overall complication profiles [9–12]. We previously reported that *orbicularis oris* muscle (OOM) fragments, obtained during cheiloplasty of patients with cleft lip (CL) patients, are a rich source of mesenchymal stem cells (MSCs) that may be useful for bone reconstruction when associated with a collagen scaffold [13].

However, up to 50% of CP cases are associated with craniofacial syndromes characterized by significant bone defects, yet occur in the *absence* of a CL deformity, where OOM is therefore not readily accessible during surgical repair of the palate. We were therefore prompted to investigate whether another regional source of muscle cells—the *levator veli palatini* muscle (LVPM)—might provide an alternative source of clinically relevant MSCs. Like OOM during cleft lip repair, LVPM can be easily obtained during planned palatoplasty in CP patients and can therefore represent an advantageous source of MSC for use in tissue engineering protocols.

Here, we describe the isolation and characterization of stromal cells from this new source, with the overarching and ultimate goal of using these cells in the surgical rehabilitation of patients with craniofacial syndromes associated with CP.

## Material and methods

Signed informed consent from all participants in this study was obtained from each patient or their legal parent or guardian(s). Study approval was granted by the Ethics Committee of the Biosciences Institute of the University of São Paulo. The laboratory experiments were carried out at Hospital Sírio-Libanês and the Human Genome Research Center in São Paulo, Brazil, and at the Regenerative Bioengineering and Repair (REBAR) Laboratory, Department of Surgery, Division of Plastic and Reconstructive Surgery at the David Geffen School of Medicine at UCLA.

**Fig. 1:**
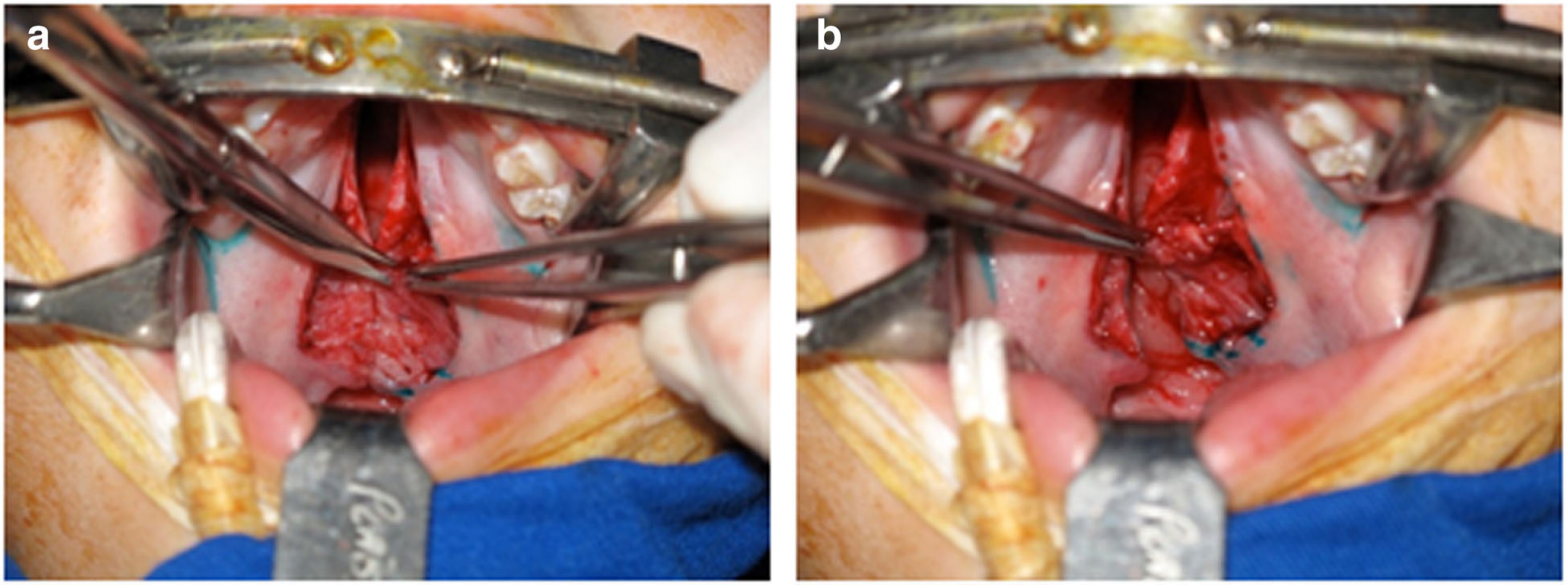
*Levator veli palatini* muscle (**a**, white arrow) and small piece *levator veli palatini* muscle harvested to obtain the cell cultures after palatoplasty (**b**, blue arrow)

LVPM fragments (*n* = 5) measuring 3–5 mm^3^ were obtained during palatoplasty in five individual CL/P patients undergoing modified von Langenbeck repair with intravelar veloplasty (Fig. 1a, b) [14]. Surgical procedures were performed at Hospital Municipal Infantil Menino Jesus, São Paulo, Brazil, and at Sobrapar Hospital, Campinas, Brazil. LVPM fragments that were harvested at the two hospitals were transported to Sírio-Libanês Hospital Laboratory.

According to local regulatory committees and pursuant to the relevant Brazilian Laws regulating advanced cell therapies (National Sanitary Vigilance Agency—ANVISA—RDC n214, February 8, 2018), all tissues were processed at the Sírio-Libanês Hospital Laboratory facilities using clean room infrastructure, air particulate control (HEPA filter) and airflow, and standard best practices for scientific investigation. These include the use of an antechamber for donning and doffing of personal protective equipment (PPE), the exclusive processing of human cells and tissues at the laboratory site, and the use of certified prion-free and apyrogenic reagents for cell isolation and cryopreservation, based on guidelines for stem cell research and the development of new clinical therapies as set forth by the International Society for Stem Cell Research (ISSR, www.issr.org).

Each muscle sample was collected in HEPES-buffered Dulbecco’s modified Eagle’s medium/Hams F-12 1:1 (DMEM/F-12; Invitrogen, Carlsbad, CA) with 200 U/mL penicillin (Invitrogen, Carlsbad, CA) and 200 μg/mL streptomycin (Invitrogen, Carlsbad, CA), kept in 4 °C, and processed within 24 h. All LVPM samples were washed twice in phosphate-buffered saline (PBS, Gibco, Invitrogen, Carlsbad, CA), finely minced with a scalpel, put inside a 15-mL centrifuge tube, and incubated in 5 mL of TrypLE Express, (Invitrogen, Carlsbad, CA) for 30 min, at 37 °C. Subsequently, supernatant was removed with a sterile transfer pipette, washed once with 7 mL of DMEM/F-12 supplemented with 10% fetal bovine serum (FBS, HyClone, Hyclone Laboratories, Logan, UT), and pelleted by centrifugation at 400×*g* for 5 min at room temperature. The pellets were resuspended and cultured in 35-mm Petri dishes (Corning, NY) containing DMEM/F-12 culture medium with 15% FBS, 2 mM l-glutamine, 2 mM non-essential amino acids, 100 U/mL penicillin, and 100 μg/mL streptomycin (Invitrogen, Carlsbad, CA). After 2 weeks, cells were washed with PBS, then dissociated in trypsin solution and seeded at 1.0 × 10^4^ cells per 25 cm^2^ for the first passage. In order to prevent cell differentiation, cultures were maintained semi-confluent and they were subcultured every 4–5 days, with medium changes every 2–3 days. After 3–4 passages, cultures yielded between 4 × 10^6^ and 8 × 10^6^ LVPMDSC.

The automated microbial detection system Bact/Alert TM 3D (Bact/Alert, BioMérieux, Durham, NC) was used to analyze the presence of aerobic and anaerobic bacteria and fungi in culture, and MycoAlertTM (MycoAlert PLUS Mycoplasma detection Kit—Lonza, Basel, Switzerland) was use for mycoplasma surveillance. Any cultures with a positive test suggesting infection were discarded.

### Flow cytometry

Flow cytometry analysis was performed by flow cytometry in a FACSCalibur flow cytometer (BD, Becton Dickinson, Franklin Lakes, NJ) and analyzed in the CellQuest program (BD, Becton Dickinson, Franklin Lakes, NJ). Cells were pelleted, resuspended in PBS (Gibco-Invitrogen, Carlsbad, CA) at a concentration of 1.0 × 10^6^ cells/mL, and stained with saturating concentration of antibodies. After a 45-min incubation in the dark at room temperature, cells were washed three times with PBS and resuspended in 0.25 mL of cold PBS. In order to analyze expression of typical cell surface markers, cells were treated with the following anti-human conjugated antibodies: CD29-PE, CD31-PE, CD34-FITC, CD44-PE, CD45-FITC, CD73-FITC, CD90-PE, CD105-FITC, and 7-amino-actinomycin D (7-AAD) staining for viability analysis (Becton Dickinson, Franklin Lakes, NJ). 7-AAD was used for the exclusion of non-viable cells in combination with PE (phycoerythrin), and FITC conjugated antibodies in flow cytometry analysis. Unstained cells were gated on forward scatter to eliminate particulate debris and clumped cells. A minimum of 5000 events were acquired for each sample.

### Mesenchymal stromal cell differentiation

To evaluate the properties of mesenchymal *stromal* cell differentiation, adherent cells (4th passage) underwent in vitro adipogenic, chondrogenic, and osteogenic differentiation according to the following protocols:

#### Adipogenic differentiation

Cells were seeded into 6-well plates (Corning Inc., Corning, NY), at a density of 2.0 × 10^5^ cells/well, in DMEM/High Glucose (Invitrogen, Carlsbad, CA), supplemented with 10% FBS (Hyclone, Hyclone Laboratories, Logan, UT), 1 μM dexamethasone, 100 μM indomethacin, 500 μM 3-isobutyl-1-methylxanthine, and 10 μg/mL insulin (all from Sigma-Aldrich, St. Louis, MO).

Fifteen days after induction, Oil Red-O (Sigma-Aldrich, St. Louis, MO) staining was used to identify intracellular accumulation of lipid-rich vacuoles [13]. Briefly, cells were fixed with 4% paraformaldehyde in PBS for 30 min, washed with PBS, and stained with a working solution of 0.16% Oil Red-O in PBS for 20 min [13].

#### Chondrogenic differentiation

Approximately 2.5 × 10^5^ cells were centrifuged in a 15-mL polystyrene tube at 400×*g* for 5 min, and the pellet was resuspended in 10 mL of basal medium. The basal medium consisted of DMEM/High Glucose (Invitrogen, Carlsbad, CA) supplemented with 1% insulin, transferrin, selenite (ITS Premix, Becton Dickinson, Franklin Lakes, NJ), 1% 100 nM dexamethasone (Sigma-Aldrich, St. Louis, MO), 1 mM sodium pyruvate (Gibco-Invitrogen, Carlsbad, CA), and 50 μM ascorbic acid-2 phosphate (Sigma-Aldrich, St. Louis, MO).

Without disrupting the pellet, cells were resuspended in 0.5 mL of chondrogenic medium, consisting of basal medium supplemented with 10 ng/mL transforming growth factor (TGF) β1 (R&D Systems, Minneapolis, MN) and 10% FBS, and maintained in a humidified atmosphere with 5% CO_2_ at 37 °C.

On day 1, tubes were gently turned over to acquire a single floating cell sphere. Medium was changed every 4 days. On day 21, samples were fixed in 10% formalin for 24 h at 4 °C and paraffin-embedded.

Cryosections (5 μm thick) were cut from the harvested micromasses and stained with toluidine blue to demonstrate extracellular matrix mucopolysaccharides [13].

#### Osteogenic differentiation

LVPM cells were cultured in osteogenic medium containing DMEM/Low Glucose (Invitrogen, Carlsbad, CA) with 0.1 μM dexamethasone and 50 μM ascorbic acid 2-phosphate. On day 9, β-glycerolphosphate (10 mM) was added to induce mineralization. On day 11, calcium content was evaluated by a Calcium Detection Assay kit (Abcam, Cambridge, UK) according to the company manual. On day 21, Alizarin Red staining was performed in order to identify accumulation of mineralized calcium. The wells were washed twice with PBS, and briefly, cells were fixed with 70% ethanol (Sigma-Aldrich, St. Louis, MO) for 30 min. After fixation, the wells were stained with 0.2% Alizarin Red S solution (pH 4.2; Sigma-Aldrich, St. Louis, MO) for 30 min. For the final wash, each well was washed with PBS (Gibco Invitrogen, Grand Island, NY) three times [15].

### Immunocompetent rat calvarial defect model

The Animal Research Ethics Committee at the University of São Paulo approved the use of Wistar immunocompetent 9-month-old male rats, body weight 320–420 g, in this experimental protocol (*n* = 5). The animals were kept in ventilated stands (Alesco, São Paulo, Brazil), in standardized air and light conditions, at a constant temperature of 22 °C with a 12-h light/day cycle. They had free access to drinking water and standard laboratory food pellets.

The animals were anesthetized with an intraperitoneal injection (0.3 mL/100 g of body weight) using a combination of ketamine hydrochloride (5%) and xylazine (2%). The heads of the rats were positioned in a cephalostat during the surgical procedure. A midline skin incision was performed from the nasofrontal area to the external occipital protuberance. The skin and underlying tissues, including the periosteum and the *temporalis* muscles, were reflected laterally to expose the full extent of the calvaria.

We next performed two symmetric full-thickness cranial defects of 4 mm diameter in size on each parietal region of the animals. The cranial defect was created with a 4-mm-diameter trephine drill, and constant irrigation with sterile physiological solution was used to prevent overheating of the bone.

The left sides (LS) of the skulls were arbitrarily selected as the control sides and were reconstructed with CellCeram™ scaffolds (Scaffdex, Finland). By comparison, the right-sided defects (RS) were reconstructed with CellCeram™ scaffolds that were seeded with 1 × 10^5^ undifferentiated LVPM stem cells. Scalps were repaired with 4–0 nylon sutures (Ethicon, São Paulo, Brazil), and the animals euthanized 30 days after cell transplantation. Calvaria were harvested for analysis at the time of euthanasia.

### Fabrication of scaffold carriers

CellCeram™ (Scaffdex, Finland) was designed in a cylindrical shape with 4 mm diameter of a bioabsorbable 60% hydroxyapatite and 40% ß-tricalciumphosphate composite with a foam-type structure of 83% average porosity, and 200–400 μm of average pore size, with an overall range of 100–800 μm. The dimensions of the scaffolds were designed to match the planned calvarial rat defects in these experiments.

### Cell preparation for transplantation procedure

We used CellCeram™ (Scaffdex, Finland) as a framework to seed 10^5^ undifferentiated LVPM stem cells and placed on a 35-mm plate (6-well plate; Corning, NY). The cells were supplemented with 2.5 mL of medium used for undifferentiated LVPM stem cells and incubated at 37 °C and 5% CO_2_ for 24 h prior to transplantation in order to adhere to the scaffold.

CellCeram™ scaffolds with adherent LVPM stem cells were transferred to the right cranial bone defect, and the cell-bearing CellCeram™ surface was positioned in direct contact with the dura mater.

### Histological preparation and quantitative analysis

The calvaria of the animals were harvested for histological assessment following euthanasia at day 30 following surgery. Tissue samples were fixed in 10% formalin solution for 24 h, decalcified in 5% formic acid for 48 h, and paraffin-embedded. For the morphological study, 5-μm sections were stained with hematoxylin and eosin (HE) and examined under a conventional light microscope.

Quantification for regenerated bone was performed using ImageJ (NIH, Bethesda, MD) with reference to the methods established in published manuscripts [16, 17]. Briefly, the split channel function was used to split the original RGB image into red, blue, and green channels. Then, the blue channel image was subtracted from the red one and the threshold range was set to 53–255, comparing to original HE images to include all regenerated bone tissue into the positive region. Then, range of interest (ROI) for all the bone defect and regenerated bone tissue was selected with the polygon selection function. Finally, the percent area of positive region in ROI which was regenerated bone was determined.

### Immunohistochemistry

The sections were deparaffinized with two 5-min washes in xylene, hydrated in graded ethanol series, and then rinsed in distilled water. For antigen retrieval, slides were incubated for 40 min in citrate buffer (95–100 °C) and then cooled for 20 min at room temperature, rinsed in PBS, and blocked for 1 h in immunofluorescent blocking buffer (IBB-5% BSA, 10% FBS, 1× PBS, and 0.1% Triton X-100). Samples were then incubated for 1 h at room temperature with 1:100 mouse anti-human nuclei monoclonal antibody (HuNu; Chemicon, Temecula, CA), washed with PBS, and incubated with secondary antibody (1:600 Alexa Fluor 594 anti-mouse IgG; Thermo Fisher Scientific) for 1 h at room temperature. Tissue was counterstained with diamidino-2-phenylindole (DAPI) (Thermo Fisher Scientific) and mounted using ProLong anti-fade (Thermo Fisher Scientific).

## Results

**Fig. 2:**
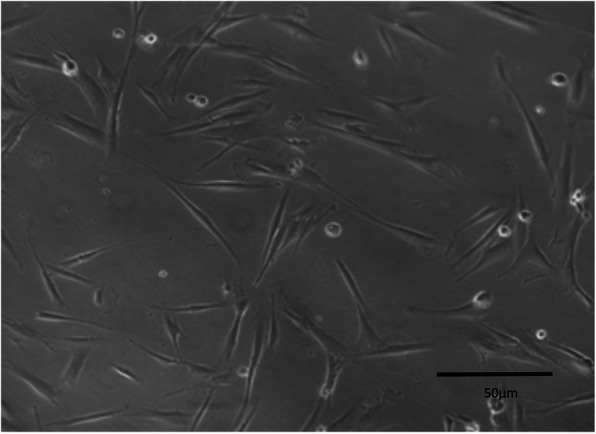
*Levator veli palatini* muscle-derived stem cell (LVPMDSC) fibroblast-like morphology. Scale bars, 50 μm

After the first enzymatic dissociation, between 5 and 7 days of culture, adherent cells were characterized by homogeneous cell layers with a MSC-like phenotype. All cell strains were successfully expanded, frozen, and thawed several times with no visible phenotypic alterations (Fig. 2).

### Flow cytometry analysis

**Fig. 3:**
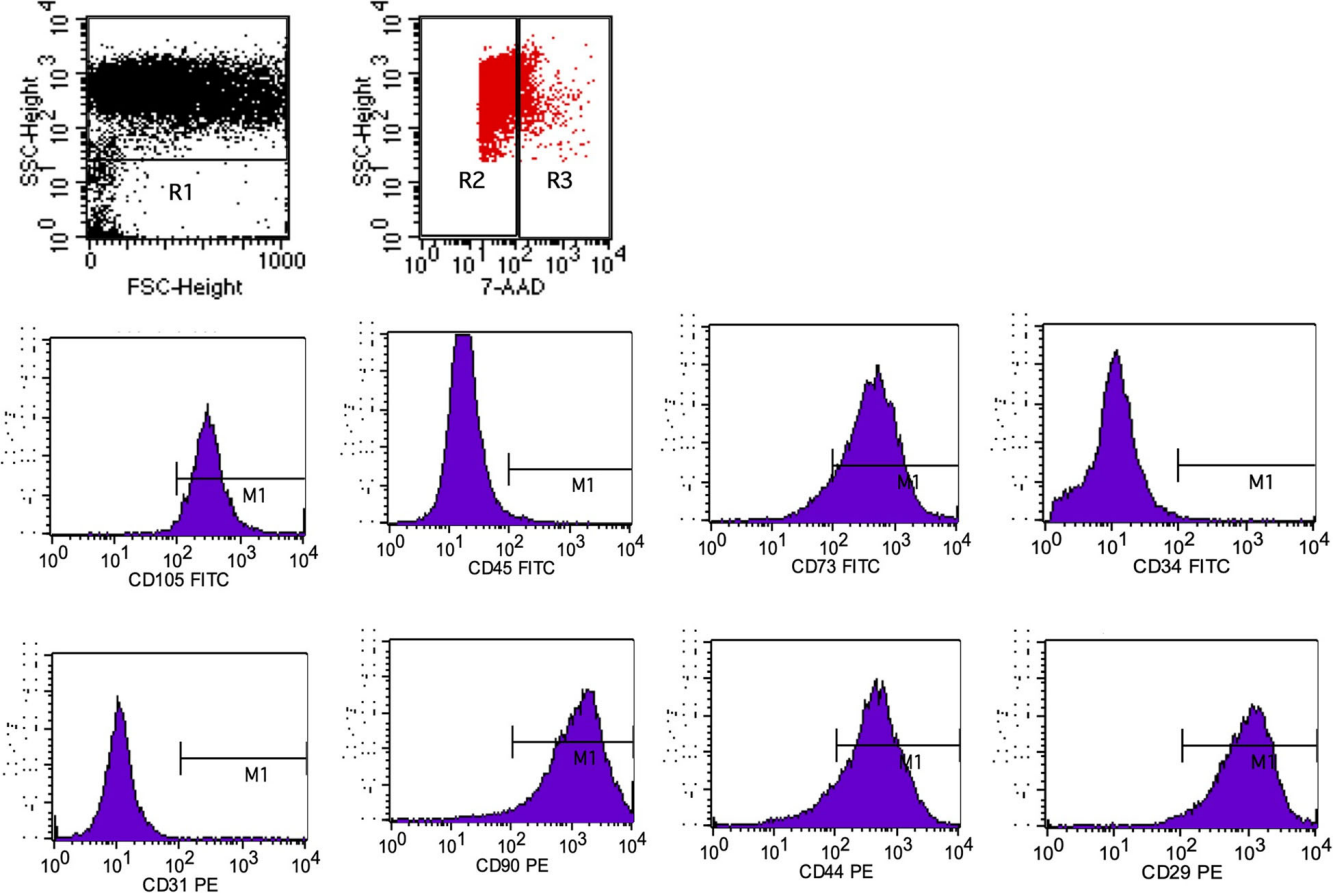
Immunophenotype analysis of LVPMDSCs. Related graphs, where it is possible to compare, for each of the 8 analyzed markers, the experimental population of LVPMDSCs selected in R1; for viability, we used 7-AAD staining (in red), where R2 = live cells (90%) and R3 = dead cells (10%). The experimental population of LVPMDSCs labeled with specific antibodies (in purple); we observe the following: negative reaction (< 2%) for endothelial marker CD31, 0.96%, and hematopoietic markers CD45, 1.14%, and CD34, 0.37%. Positive reaction was observed for the adhesion markers CD29, 99.82% (C); CD 90, 98.06%; and CD44, 90%, and for the mesenchymal markers CD73, 99.66%, and CD105, 97.78%. CD, cluster of differentiation

None of the 5 LVPM cell populations expressed the CD34 and CD45 hematopoietic lineage marker or the CD31 endothelial marker. The majority of cells expressed high levels of adhesion markers (CD29, CD44, and CD90) and MSC markers (CD73 and CD105) (Fig. 3; Table 1). These results indicate that the cells obtained from LVPM were mesenchymal in nature. The LVPM cells have shown more 90% the live cells.

**Table 1:** Percentage of positive reactivity for each cell strain and for each cell marker used on flow cytometry experiment

Marker	F3440-1 (%)	F3404-1 (%)	F3420-1 (%)	F3492-1 (%)	F3436-1 (%)
CD29	99.82	95.85	95.82	99.64	98.59
CD73	99.66	98.82	98.82	99.04	98.87
CD90	98.06	97.88	97.88	99.78	97.78
CD105	97.78	94.00	93.24	90.42	89.34
CD44	90	89.8	92.5	91.78	90
CD31	0.96	0.80	0.80	0.76	0.97
CD45	1.14	0.64	0.64	1.18	1.18
CD34	0.37	0.4	0.08	0.1	0.2
7-AAD	90	96	92	96	98

### Multilineage differentiation

Multilineage differentiation was performed for 5 independent samples of LVPM cells. No obvious qualitative differences in their differentiation potential were observed.

**Fig. 4:**
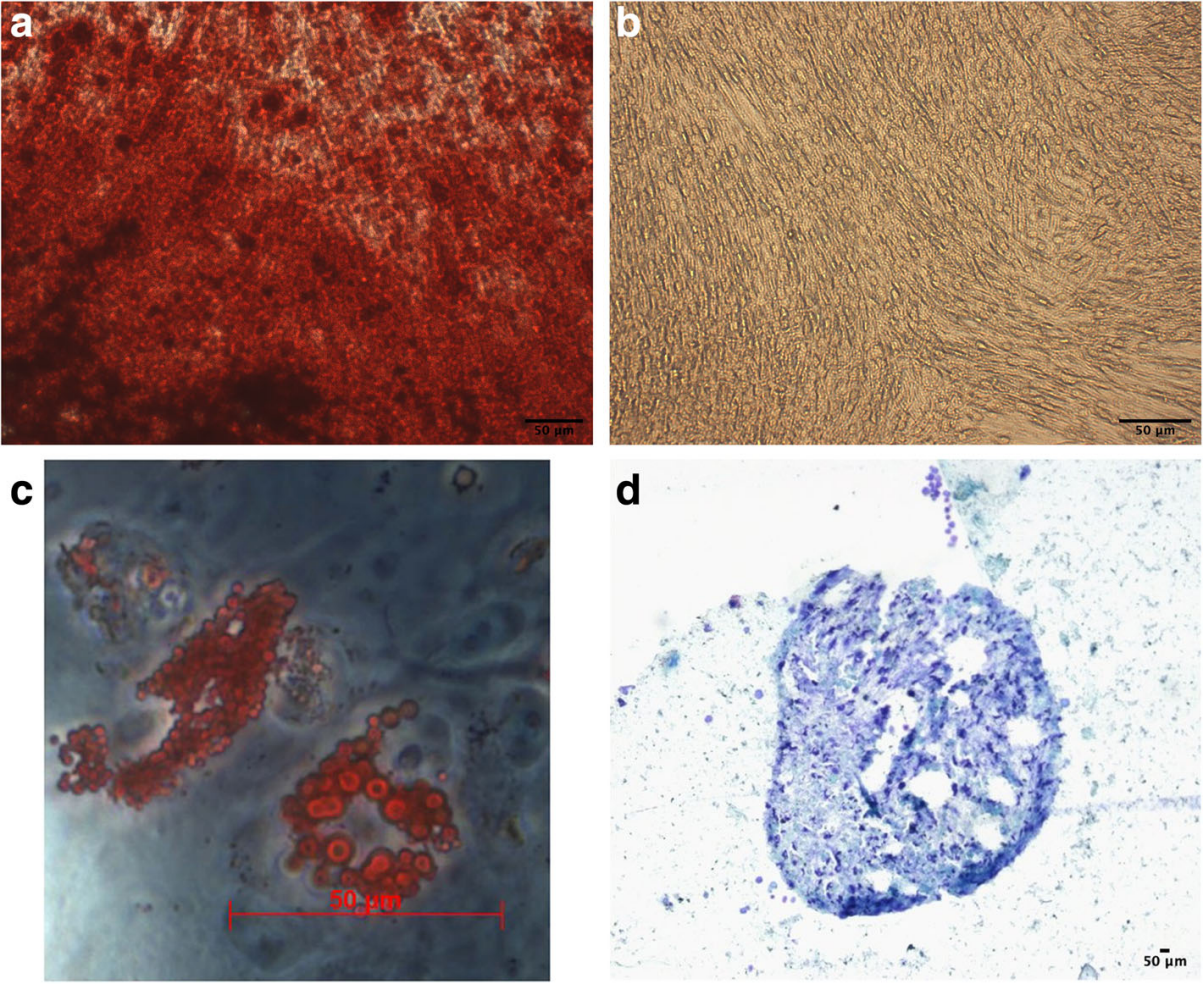
In vitro staining of LVPMDSCs. Osteogenic differentiation: Alizarin Red S staining revealing calcified extracellular matrix 21 days after osteogenic induction, white arrows shows the calcium deposition (**a**) and its negative control (**b**). Adipogenic differentiation: Oil Red-O staining (**c**). Chondrogenic differentiation: toluidine blue staining (**d**). Scale bars, 50 μm

**Fig. 5:**
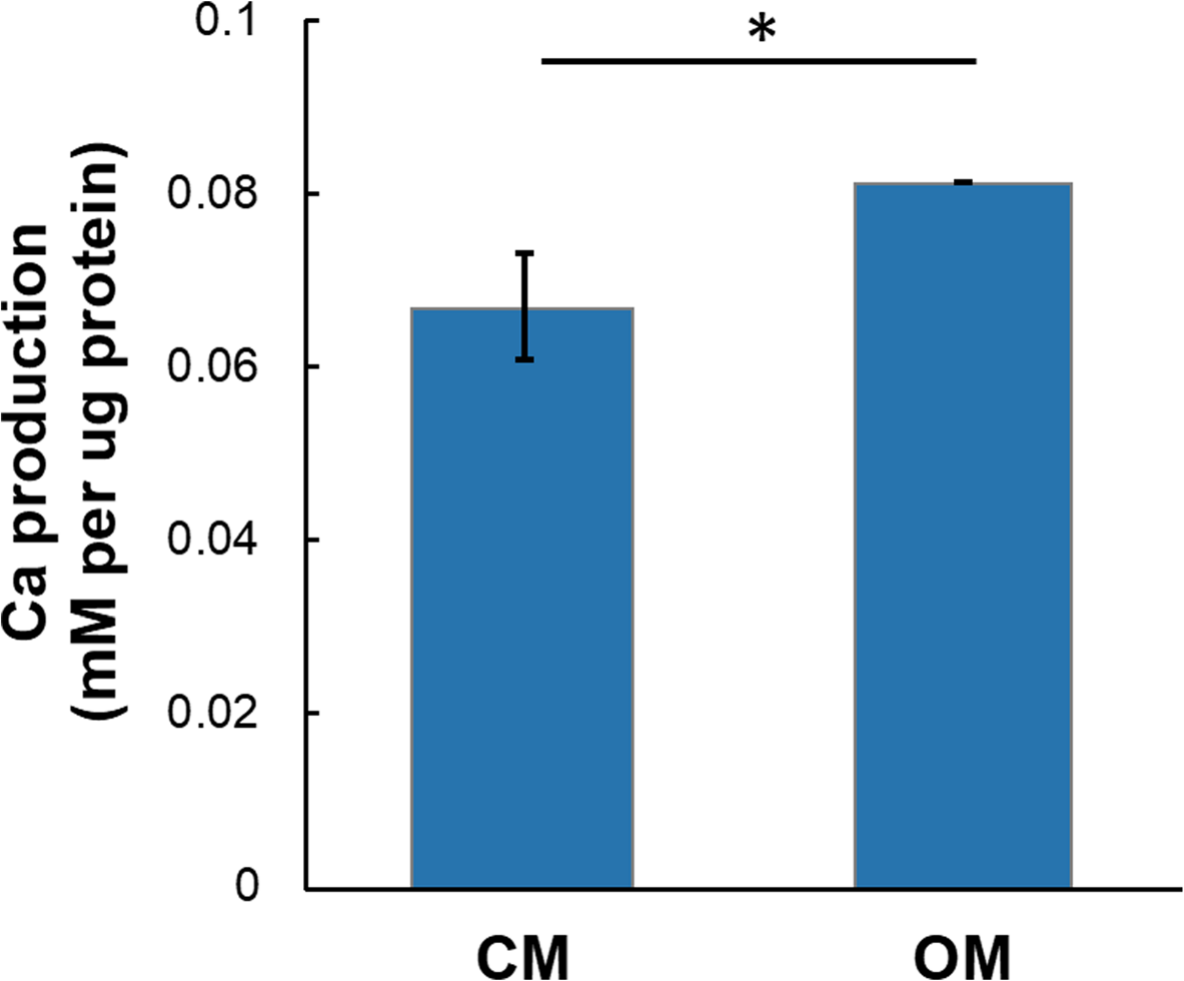
Cells were cultured with control medium (CM) or osteogenic medium (OM). After 11 days treatment, calcium content was evaluated using a kit. Cells treated with OM showed significantly increased calcium production compared with the cells in CM. *p < 0.05 by the Student’s *t* test

The plasticity of adherent cells obtained from LVPM was assessed 3 weeks after in vitro induction of osteogenic and chondrogenic differentiation. The adipogenic differentiation was observed after 15 days. The LVPM cells from all 5 strains were able to undergo chondrogenic, adipogenic, and osteogenic differentiation in vitro (Fig. 4). Together, these results confirmed the mesenchymal stromal nature of the isolated cells, as well as their multipotency. After 11 days with osteogenic induction, we can observe calcium production in LVPM induced with osteogenic medium (Fig. 5). Based on this observation, we renamed the cells isolated from LVPM as *levator veli palatini* muscle-derived stromal cells (LVPMDSCs).

### In vivo osteogenic potential of LVPMDSC

The in vivo osteogenic potential of LVPMDSCs was assessed in a calvarial defect model in non-immunosuppressed Wistar rats. None of the experimental animals died of infection, nor any other complication as a result of surgery or the cell/scaffold transplantation process.

**Fig. 6:**
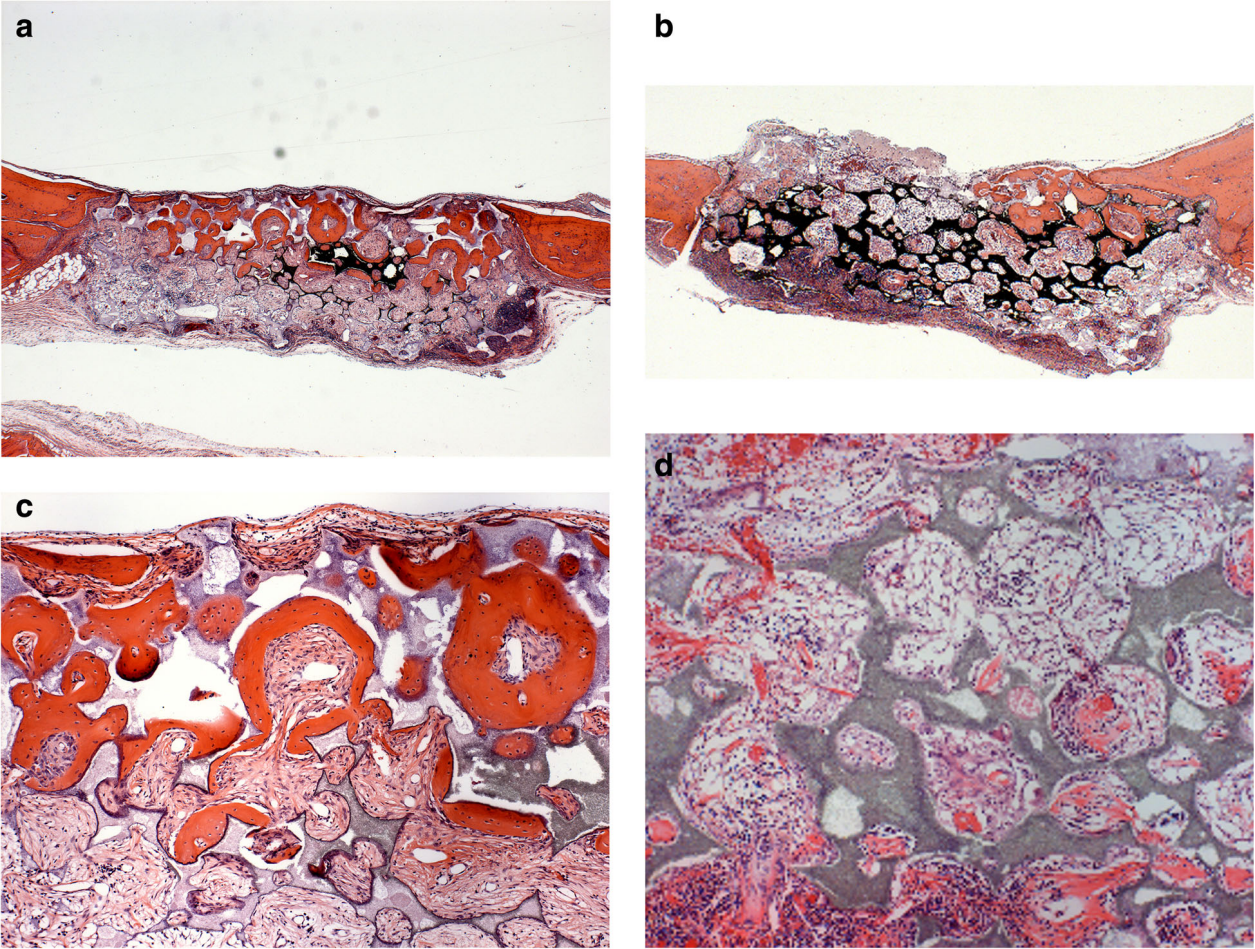
Histological analysis of bone neoformation at 30 days post-surgery. Rat defect (right side) seeded with LVPMDSC associated with CellCeram™, revealing higher bone neoformation in different magnifications—× 25 and × 100 (**a**, **c**), when compared with the defect (left side) where only CellCeram™ was applied (**b**, **d**). In **a** and **c**, we observe greater bone neoformation (blue arrows) intermixed with granulation tissue with few remnants of CellCeram™ (yellow arrow), while in **b** and **d** the defect is filled with loose connective tissue exhibiting chronic inflammatory infiltrate (II), intermingled with remnants of CellCeram™ (yellow arrows) and only a small number of trabeculae of recently formed bone. NB, naive bone; CD, critical defect; II, inflammatory infiltrate

Histological examination of the cranial defect 30 days following surgery revealed significantly new bone formation on the RS (scaffold + LVPMDSC) compared to the LS (acellular scaffold) (Fig. 6a, b). The trabeculae of the newly formed bone (woven bone) observed in the RS defects were intermixed with granulation tissue and with remnants of the CellCeram™ biomaterial. By comparison, the LS defects healed with loose connective tissue exhibiting chronic inflammatory infiltrates, intermingled with larger amounts of scaffold remnant (Fig. 6c, d).

**Fig. 7:**
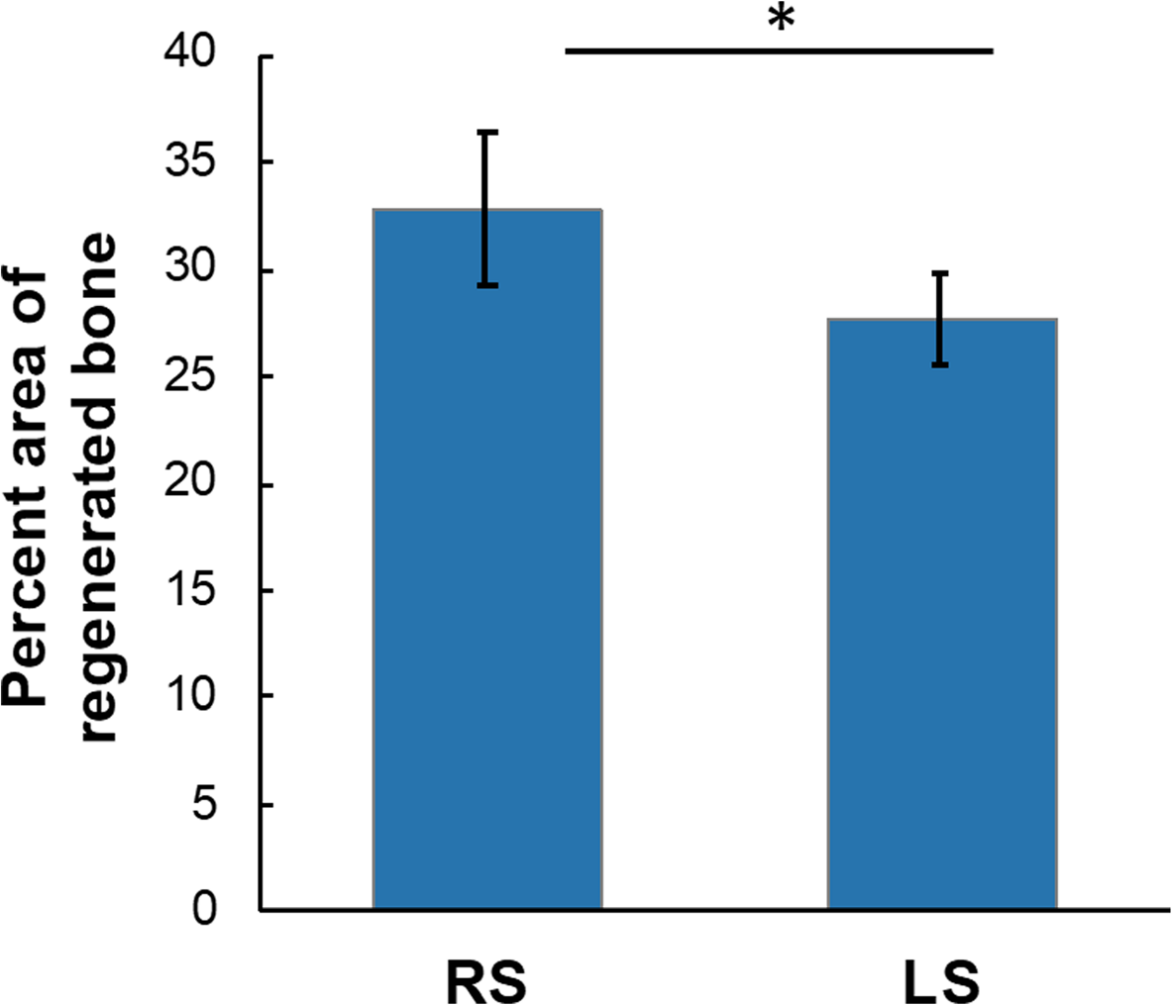
Regenerated bone was quantitatively measured by ImageJ. The percent area of regenerated bone by CellCeram™ + LVPMDSC (RS) was significantly greater than that by CellCeram™ (LS). *p < 0.05 by the Student’s *t* test

The regenerated bone area quantitatively measured by ImageJ showed the percent area of regenerated bone by CellCeram™ + LVPMDSC (RS) was significantly greater than that by Cell Ceram™ (LS), *p* < 0.05 by Student’s *t* test (Fig. 7).

**Fig. 8:**
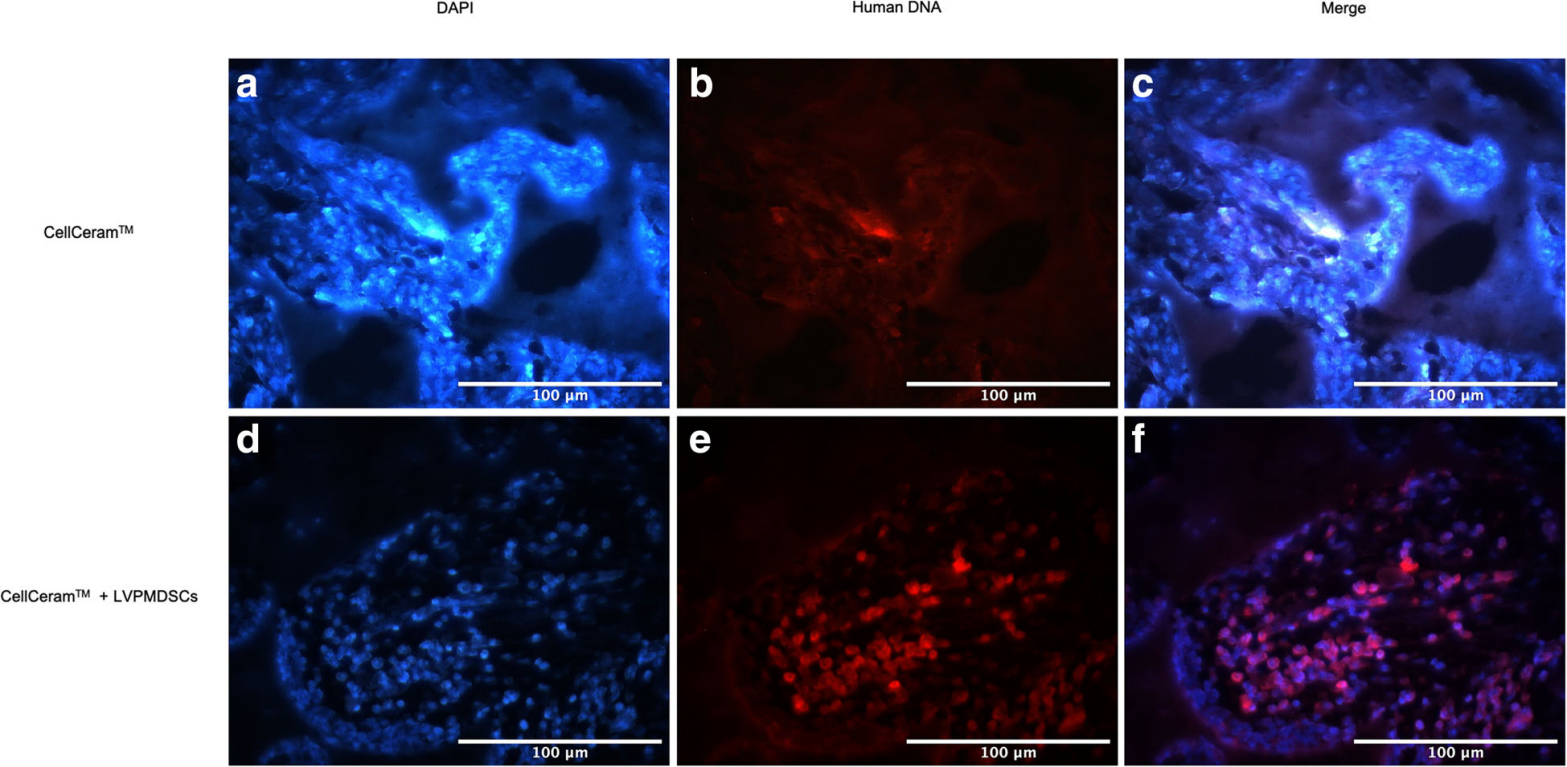
Immunofluorescence analyses of human remaining cells at 30 days post-surgery. Rat defect (left side) where only CellCeram™ was used (**a**–**c**) showed only the presence of rat cells in blue (DAPI) on the slides. In the rat defect (right side) seeded with LVPMDSC associated with CellCeram™ (**d**–**f**), we observe rat cells (in blue DAPI) (**a**) and human remaining cells (human nuclei—red fluorescent staining) in the inflammatory infiltrate regions (**b**) and the overlap of DAPI and human nuclei (**c**) showing the presence of the human cells in the inflammatory tissue. Scale bars,100 μm

We also observed positive staining for human nuclei thorough immunohistochemical analysis only on the inflammatory tissue on the RS where the LVPMDSCs were added to the CellCeram™ biomaterial. On the LS (acellular scaffold reconstruction), we observed positive reaction only for DAPI staining, without any evidence of human cell proliferation (Fig. 8 and supplementary material Fig. 1).

## Discussion

Initially defined as bone marrow precursors, new evidence suggests that MSCs are present in virtually all organs, possibly playing an important role in tissue maintenance and regeneration [12, 13, 18, 19]. The possibility of using MSCs in regenerative medicine protocols has opened a new field of investigation aiming to find the best sources for obtaining multipotent stem cells, with a specific focus on cells that can be obtained in non- or minimally invasive ways.

In this study, we have demonstrated that LVPM fragments, which can easily be obtained in patients undergoing palatoplasty, represent a novel source of multipotent MSCs. We have therefore begun to refer to these cells, including herein, as levator veli palatini muscle-derived stromal cells (LVPMDSCs).

LVPMDSCs have similar characteristics to the *orbicularis oris* muscle-derived stem cells (OOMDSCs) obtained from cleft lip and palate patients, which we have previously described [13, 15]. The similarity between them resides in their immunophenotype: like OOMDSCs, LVPMDSCs were strongly positive for mesenchymal and adhesion cell surface markers, but did not demonstrate the presence of endothelial or hematopoietic markers [13, 15].

From a technical standpoint, our results show that the pre-plating technique used here can be applied to isolate stromal cells with similar properties from two distinct sources: *orbicularis oris* and *levator veli palatini* muscles. Whether this can be generalized to cells derived from other muscles remains to be investigated, since markers used for cell characterization vary across studies [20, 21].

The mesenchymal/multipotential nature of isolated LVPMDSCs in this study was confirmed by the high expression of adhesion and MSC markers in these cells, along with their demonstrated multilineage differentiation potential [22, 23]. These phenotypic hallmarks were similar to the ones seen in primary MSCs obtained from other sources, such as fat, dental pulp, bone marrow, fallopian tube, orbicular oris muscle, and umbilical cord vein in other studies conducted by our team [13, 24–26].

Such features of LVPMDSCs, in conjunction with their capacity to increase bone formation in vivo when associated with CellCeram™ scaffolds, indicate that this cell type has the potential for clinical application, especially in bone tissue engineering protocols being developed to treat complex craniofacial malformations. Our data showed that LVPMDSCs seeded onto CellCeram™ scaffolds lead to greater amounts of bone regeneration compared to acellular scaffolds, showing the osteogenic potential of these cells in vivo. Our results are consistent with other studies that have demonstrated how MSCs of alternate origin, when seeded onto biocompatible scaffolds, also lead to higher levels of new bone formation when compared to the use of acellular scaffolds [13, 24, 27, 28]. In a study by Gamblin et al. [29], calcium phosphate granules were implanted in the nude mice muscles with or without human MSC and the chronological events leading to osteoinduction were evaluated. They reported that hMSC induced an early mobilization of circulating monocytes to the implantation site as the presence of macrophages and osteoclasts was significantly upregulated, suggesting their implication in the mechanism of bone formation. They also concluded that human MSC did not participate directly in osteogenesis; rather, they increased the innate immune response and enabled to speed up the mobilization of monocytes to the implantation site, and an upregulation of osteoclasts and macrophages at the implantation site was correlated with increased bone formation due to human MSC associated to calcium phosphate particles. In our study, it is important to highlight that in our in vivo calvaria reconstruction experiments, a positive reaction for human nuclei antibody was observed in defects reconstructed with LVPMDSCs seeded onto CellCeram™ scaffolds. In these defects, new bone formation was observed in the middle of the defects while human nuclei were identified in the marginal areas of inflammation. This observation supports the notion that the LPVMDSCs have a paracrine function that may stimulate bone formation within the defect. By comparison, acellular scaffold reconstruction resulted in new bone deposition primarily along the margins of the defect with a paucity of central defect osteogenesis.

Different types of biocompatible scaffolds have been used in tissue engineering research. These include collagen membranes [10, 13], hydroxyapatite [12, 27], and calcium phosphate [30]. Here, we suggest that CellCeram™, a biomaterial composed of hydroxyapatite and ß-tricalciumphosphate, is an effective alternative in this tissue engineering paradigm, as its size and three-dimensional shape can be custom-synthesized. This enables each CellCeram™ scaffold to be individually designed according to the precise anatomical requirements that define any specific bone defect.

We observed no post-surgical complications, such as wound infection or dehiscence, graft rejection, or any other overt sign of gross inflammation. The fact that immunocompetent animals were used in this study, and that these animals underwent xenotransplantation of human multipotent MSCs, suggests that LVPMDSCs helped mitigate an anticipated immunological response in such an experimental setting. This result is consistent with similar prior observations with other types of MSCs, in three previous works by our group [13, 24, 31]. Moreover, it has been reported that MSCs possess immunomodulatory properties [27]. Collectively, these findings suggest that heterologous LVPMDSCs may safely be used in clinical bone tissue engineering protocols without elevated risk of immunologic-mediated inflammatory responses.

Based on our previous experience in clinical trials using deciduous dental pulp stem cells associated with biocompatible scaffolds (Bio-Oss Collagen®^,^ Geistlish) to reconstruct alveolar clefts (Clinicaltrials.gov: NCT01932164 and NCT03766217), we believe that it will be possible to scale expansion of LVPMDSCs and seed these cells onto custom three-dimensional printed scaffolds as the foundation of a clinically applicable bone tissue engineering strategy [12]. Because LVPMDSCs and deciduous dental pulp stem cells have similar in vitro and in vivo properties, we are convinced that these two distinct yet related cell populations will behave similarly in the setting of major bone reconstructive challenges.

Like the protocol that was implemented in our autologous dental pulp stem cell alveolar cleft reconstruction clinical trial [12], we intend to collect levator veli palatine muscle samples during the palatoplasty surgery, send the samples to the laboratory, isolate the LVPMDS, seed them onto scaffolds, and return a “Bioengineering Kit” (composed of the LVPMDSC/scaffold combination) back to the operating room for implantation. The same approach can be used in the future for heterologous grafts. The use of this bioengineering “kit” can reduce morbidity by eliminating the need to for autogenous grafts and separate donor site surgery. Surgeons who treat congenital craniofacial differences always try to decrease the number of operations in children requiring complex or staged reconstruction [10]. The potential to minimize the numbers of required surgeries and, by extension, surgical morbidity by using MSCs seeded onto various scaffolds to generate new bone formation is an exciting prospect [10–12].

## Conclusion

In summary, our study suggests that, in the future, LVPMDSCs associated with CellCeram™ scaffolds may be used as a substitute for autologous bone grafting in craniofacial syndromes, specifically those that include cleft palate in their phenotype. In patients with Treacher-Collins, Goldenhar, or other syndromes that may use distraction and/or bone grafting procedures, autologous LVPMDSCs in association with CellCeram™ may provide a viable clinical alternative. The isolation and characterization of LVPMDSCs open new opportunities for the use of these cells in bone reconstruction.

To our knowledge, this is the first study to describe the isolation, in vitro expansion, and multilineage differentiation potential of mesenchymal stromal cells derived from *levator veli palatini* muscle. These cells enhance bone regeneration in vivo when associated with CellCeram™. Thus, our results suggest that these cells are suitable for future applications in bone tissue engineering for craniofacial diseases.

## Supplementary Information

**Additional file 1.** Supplementary Materials.

## Data Availability

The corresponding author had full access to all the data in the study and had final responsibility for the decision to submit for publication. Please contact author for data requests.

## Abbreviations

OOM: Orbicularis oris muscle
CL/P: Cleft lip and palate
MSCs: Mesenchymal stem cells
LVPM: Levator veli palatini muscle
GMP: Good practices of manipulation
ANVISA: Agência Nacional de Vigilância Sanitária, Brazil (Brazilian National Sanitary Vigilance Agency)
DMEM/F-12: Dulbecco’s modified Eagle’s medium/HAMs F-12
FBS: Fetal bovine serum
PBS: Phosphate-buffered saline
LVPMDSCs: Levator veli palatine muscle-derived stem cells
