# Supplementary material for: Human levator veli palatini muscle: a novel source of mesenchymal stromal cells for use in the rehabilitation of patients with congenital craniofacial malformations
Source: Stem Cell Res Ther. 2020 Nov 25;11:501. doi: 10.1186/s13287-020-02017-7 (PMC7687766; doi:10.1186/s13287-020-02017-7)
Supplement: Supplementary file 1 — Additional file 1. Supplementary Materials. [file 13287_2020_2017_MOESM1_ESM.docx]

Supplementary Materials

Figure 1. Immunofluorescence analyses of human remaining cells at 30 days post-surgery. Rat defect defect (left side) where only CellCeram^TM^ (A), showed only rat cells (green arrowin blue DAPI). In the Rat defect (right side) seeded with LVPMDSC associated with CellCeram^TM^ (B), we observe rat cells (in blue DAPI) and human remaining cells (human DNA - red fluorescent staining) in the inflammatory infiltrate regions (yellow arrow); Bone neoformation (white arrow) and only rat cells (green arrow – in blue DAPI). Scale bars:100 µm.


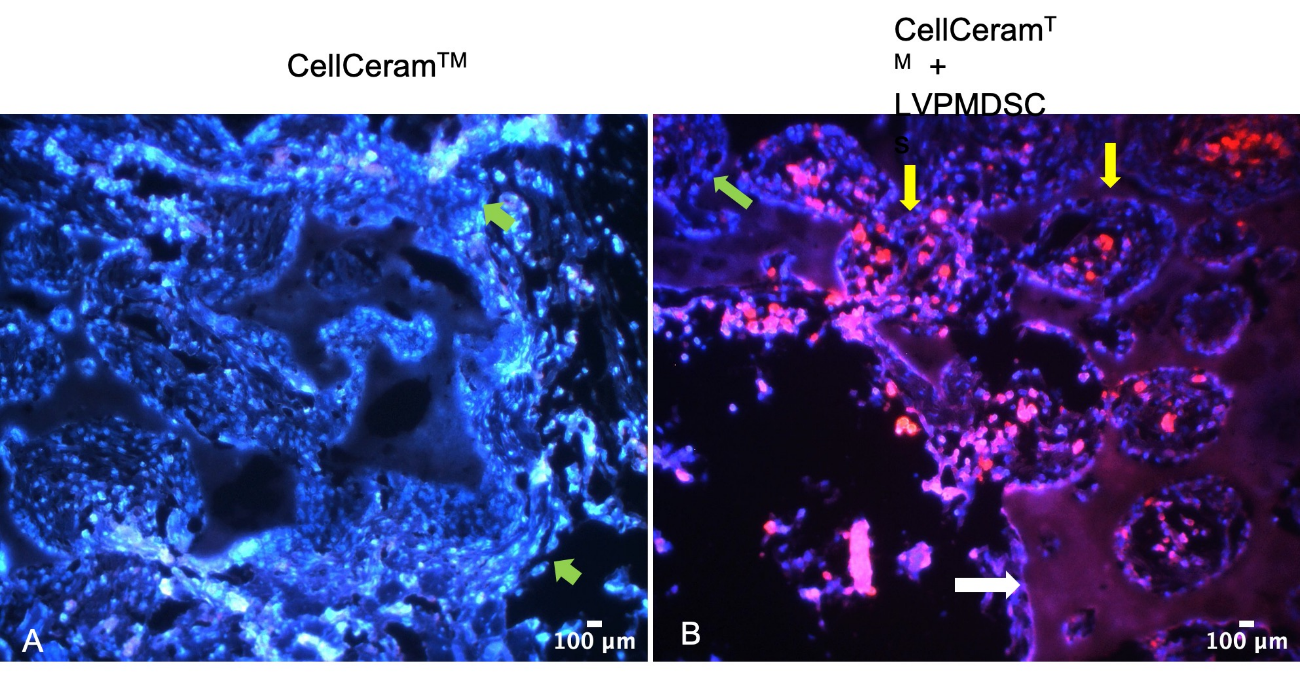


Figure2. The proliferation assay of two patients that have both muscle (orbicular oris and levator veli palatine muscle) to compare the proliferation potential of LVPMDSC with OOMDSC (orbicular oris muscle derived stem cells). We obtained the OOMDSC from cleft lip and palate patients during the Cheiloplasty and we obtained the LVPMDSC from the same patients during the palatoplasty surgery. The proliferation results were very similar (Person’ s test correlation: Patient 1 = 0,9 Patient 2= 0,8).
